# Key Risk Factors Affecting Farmers’ Mental Health: A Systematic Review

**DOI:** 10.3390/ijerph16234849

**Published:** 2019-12-02

**Authors:** Sahar Daghagh Yazd, Sarah Ann Wheeler, Alec Zuo

**Affiliations:** Centre for Global Food and Resources, Faculty of Professions, University of Adelaide, Adelaide, SA 5000, Australia; sahar.daghaghyazd@adelaide.edu.au (S.D.Y.); alec.zuo@adelaide.edu.au (A.Z.)

**Keywords:** farmers’ mental health, farming stress, mental disorder, systematic review

## Abstract

Recently, concern has increased globally over farmers’ mental health issues. We present a systematic review of the outcomes, locations, study designs, and methods of current studies on farmers’ mental health. In particular, this review aims to fill an important gap in understanding of the potential key risk factors affecting farmers’ mental health around the world. 167 articles on farmer mental health were included in a final systematic review using a standardized electronic literature search strategy and PRISMA guidelines. The four most-cited influences on farmers’ mental health in the reviewed literature respectively were pesticide exposure, financial difficulties, climate variabilities/drought, and poor physical health/past injuries. The majority of studies were from developed countries, most specifically from the United States, Australia, and the United Kingdom. Comparative studies on the mental health of farmers and other occupational workers showed mixed results, with a larger portion identifying that psychological health disturbances were more common in farmers and farm-workers. Knowledge of farmer psychological disorder risk factors and its impacts are essential for reducing the burden of mental illness. Further research will be required on climate change impacts, developing country farmers’ mental health, and information on how to reduce help-seeking barriers amongst farmers.

## 1. Introduction

Researchers have identified a number of occupational health risks through studies of farming communities, and some have specified farming as an especially stressful occupation [1,2,3,4]. Farming is associated with a range of physical and mental health risks because of the hard work under challenging conditions [2]. Studies on mental health in farming communities around the world have identified several common risk factors, namely: commodity prices, debt, climate change, drought, overwork, government regulations, isolation, role conflict, time pressure, and poor housing [5,6,7,8,9,10,11,12,13,14,15,16,17,18,19,20,21,22,23,24,25,26].

It has been shown that chronic stressors have a major influence on well-being and health. Particularly, stress is associated with an increased prevalence of mental disorders, such as depression and anxiety [7]. Stress has dominated the literature as one of the most broadly researched psychosocial constructs, mainly in the work-related stress area. Work-related stress is defined as a conflict when the demands of work are high, and the worker cannot manage, control, or cope with that stress [27]. For farmers in particular, the advent of future climate change means that their job will become even more stressful [28]. Williams [29] reported that chronic stress among farming communities might lead to physical problems (e.g., headaches, sleep problems), mental problems (e.g., anxiety, anger, depression), and cognitive issues (e.g., memory loss, inability to make decisions). Farmers have also been more likely to report that life was not worth living than non-farmers [2]. Mental problems among farmers can affect their lives in different ways, and the impact of stress factors are varied among them. These include less interest in pleasure, less concentration, loss of appetite, weight change, tiredness, irritability, problems sleeping, fatigue, loss of control, and anxiety [5,28,30,31,32,33,34]. Also, loss of self-esteem, withdrawal from social activity, relationship breakdown, forgetfulness, loss of temper, relaxation problems, feeling blue, and substance abuse have been reported [9,13,35,36]. A danger of burnout and exhaustion is possible with all these symptoms. Burnout is a gradually developing disorder that may consist of physical and mental exhaustion, a cynical attitude towards work, and a reduction in self-esteem [37]. Most importantly, mental disorders have been identified as one of the key risk factors for suicide attempts among farmers [34]. High suicide rates among farmers, farm manager and agricultural labourer have been reported in several studies [38,39,40,41], which is considered one of the most serious concerns affecting some farming communities. As the issue of farmers’ mental health raises many concerns, we conducted a search of the literature to answer the following main research questions: what are the key risk factors affecting farmers’ mental health and how does this differ around the world?

To explore the research question, this paper systematically reviews published studies on farmers’ mental health, and details the risk factors that have been considered and how farmer mental health has been measured. What is clear is that interpretations of mental health outcomes vary across identified studies, and most of the times outcomes are not clearly defined. We distinguish between mental health and mental disorder here. According to the WHO (2007; p. 1), mental health is: “a state of well-being in which the individual realizes his or her own abilities, can cope with the normal stresses of life, can work productively and fruitfully, and is able to make a contribution to his or her community” [42]. Mental disorders are normally defined by some combination of abnormal thoughts, emotion, behaviour, and relationships with others [43]. Mental disorders include depression, anxiety, stress, schizophrenia, bipolar disorder, and emotional/psychological distress [44]. The most common mental disorders are said to be anxiety and depressive disorders, which are a reaction to the stresses of life. A person with an anxiety disorder feels distressed a lot of the time, for no apparent reason, and a person with a depressive disorder can experience a long-term depressed mood and loss of interest in activities that used to be enjoyable [45]. The burden of mental disorders continues to grow with substantial impacts on health and major social, human rights, and economic consequences around the world [44]. 

Given the growing farming pressures in many countries (e.g., declining productivity, declining terms of trade, worsening weather impacts, and deteriorating soil and water quality), evidence-based understanding of risk factors on farmer mental health will become increasingly more important to improve the efficiency of prevention efforts. Hence, we sought to understand what the potential key risks affecting farmers’ mental health are, as well as if these risks vary across space and time. 

## 2. Materials and Methods

This systematic review followed the standard Preferred Reporting Items for Systematic Reviews and Meta-Analyses (PRISMA) [46], namely: (1) identification of literature; (2) screening questions; (3) eligibility using inclusion criteria; and (4) assessment of the quality of the studies and detection of any possible bias, which are discussed in the following sections.

### 2.1. Identification

To identify relevant literature, we searched the literature published until April 2019 in electronic databases PsycINFO, PubMed, Scopus and Google Scholar using the following keywords: “Mental health” OR “mental disorder” OR “depression” OR “distress” OR “anxiety” OR “stressors” in the combination of “farmer” “farmworker” “agricultural worker”. Note: suicide and suicide ideation was not included in this review.

### 2.2. Screening Questions

The electronic database search generated 1224 English language articles (excluding duplicates), and after screening the title and abstract, 436 studies were included in the review. Then the body of these selected articles were screened with the following questions:

1. Are farmers included as a general study population? (y/n)
2. Are any kind of “mental disorders” part of the study? (y/n)

Based on the results of the screening questions, 329 studies were first included in the review. Studies were excluded because of limited relevance to farmers’ mental health issues (e.g., those focused on rural communities as a whole or those related to farmer/rural suicidal behaviours only). 

### 2.3. Eligibility Assessment

The following inclusion criteria were then applied:

1. Does the study clearly mention which risk factors/stressors affect farmers’ mental health (y/n)?
2. Does the study detail the direction on farmers’ mental health (+/−/0)?

**Figure 1:**
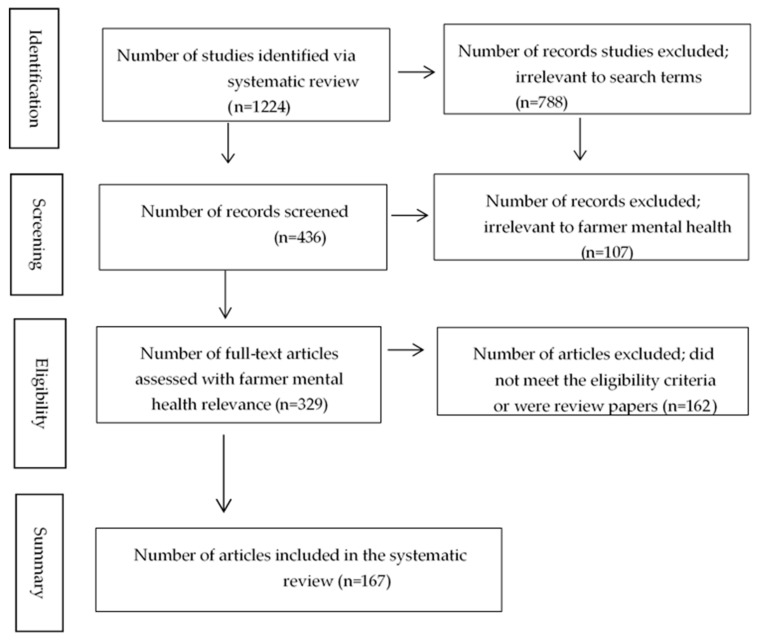
Schematic for identifying studies.

Among the identified articles, 162 failed to meet the eligibility criteria, hence 167 articles were included in the review. Of the 167 articles, 146 of these were quantitative studies. Figure 1 provides the roadmap followed for the studies selection. 

### 2.4. Assessment of the Quality of the Studies and Detection of Possible Bias

Each study that used quantitative methods was rated using the OHAT risk of bias rating tool [47]. The OHAT risk of bias tool consists of a set of questions to address the main bias domains (see Appendix A for the bias questions). Each question within the tool receives one of four assessment levels (definitely low risk of bias, probably low risk of bias, probably high risk of bias, and definitely high risk of bias). Based on the answers to the assessment questions, each study was classified into one of the three tiers proposed by the OHAT to synthesise risk of bias evaluations across studies. 

NVivo, a qualitative data analysis software, was used to classify the total 167 selected studies on farmer mental health. We provide an overview of the findings of this review for: (i) study geographical focus, (ii) mental health scales or measures, (iii) mental health of farmers versus non-farmers, (iv) key farm risk factors, (v) socio-demographic characteristics of farmers with poorer mental health, and (vi) farmer help seeking behaviour.

## 3. Systematic Review Results

### 3.1. Geographic Focus 

**Figure 2:**
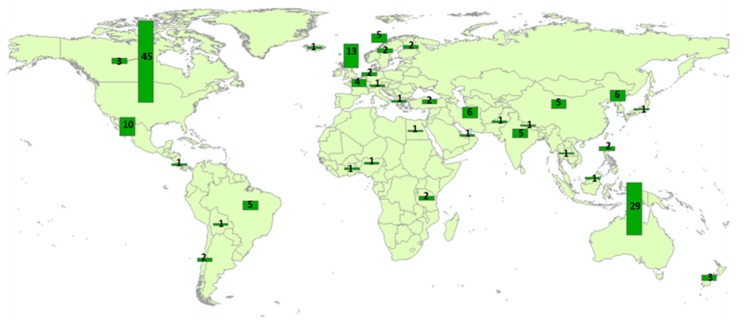
Number of farmers’ mental health studies published by country.

Research into farmer mental health has been conducted in several countries, but mostly in developed countries. As Table 1 and Figure 2 show, the United States, Australia and the United Kingdom have conducted the greatest amount of research in this space (27%, 17%, and 8% respectively). Of the papers that focused on farmers’ mental health, the majority used quantitative methods (146 studies) versus qualitative methods (18), versus a combination of both qualitative and quantitative (3). 

**Figure 3:**
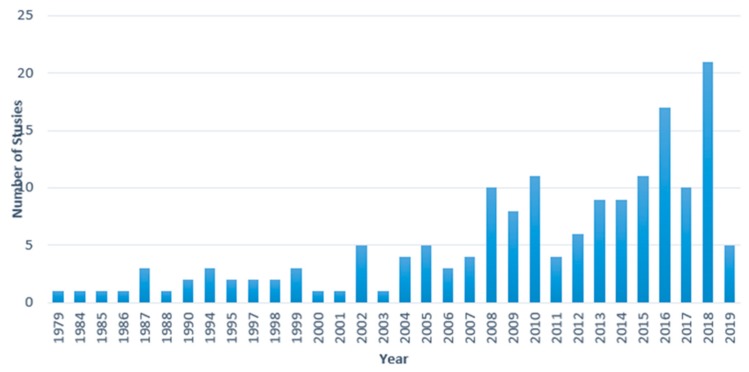
Number of farmers’ mental health studies from 1979 to April 2019.

Figure 3 shows an increasing focus on farmer mental health research over the past couple of decades, with an increase from the mid-2000s onwards. This increase was driven by a surge in research by researchers on Australian farmers’ mental health 2005 onwards (with was near the middle of the Millennium Drought in Australia). The highest number of publications was in 2018.

### 3.2. Measures and Methods of Farmer Mental Health and Assessment of Quality

Method and measurement assessment of farmer mental illness has varied greatly (see Table 2). Our systematic review indicates that literature used a variety of methods and scales for measuring farmer mental health. The most common method for measuring mental health was the Centre for Epidemiologic Studies-Depression Scale. However, other reasonably common methods such as Kessler 10, Hospital Anxiety and Depression, Clinical Tests, Mini-Mental State Examination, and SF-36. 

Several measures have also been constructed to measure agricultural-related stress, including the Farm/Ranch Stress Scale, the Edinburgh Farming Stress Inventory, Welke’s Farm Ranch Stress Inventory, and Migrant Farmworker Stress Inventory.

The assessment of bias by using the tiering approach across the studies showed that, out of the 146 quantitative studies, 99 (68%) of them were categorised in “Tier1” or “plausible bias unlikely to seriously alter the results”; 37 (25%) of studies were categorised in “Tier2” or “plausible bias that raises some doubt about the results”; and only 10 studies (7%) of studies were categorised in “Tier3” or “plausible bias that seriously weakens confidence in the results.”

### 3.3. Mental Disorders among Farmers Versus Non-Farmers

The systematic review found that 28 articles (17% of the total) compared farmers’ mental health with other occupational groups. Out of those articles, 20 studies (71%) suggested farmers have worse mental health issues than the general population, e.g., [86,87,88]. Also, the National Institute for Occupational Safety and Health [89] examined 130 different occupations and found farm workers and farm owners had the highest rate of deaths due to stress-related conditions and mental disorders. Higher mental disorder levels and poorer vitality have been reported for animal farmers [90,91] and dairy farmers [90,92], compared to non-farmers. Others also find a higher prevalence of mental disorders in farm workers as compared to employed non-farmworkers and other occupations such as teaching, office work, and building construction [62,86,90,93,94,95,96,97,98,99,100,101]. Wheeler et al. [26] identified that some Australian irrigators experienced high levels of psychological distress nationally; higher than dryland farmers or the Australian population. Gevaert et al. [85] found farmers, self-employed, and own account workers have worse mental health compared to medium-to-large employers and liberal professions. Ulrich et al. [102] identified that stress was significantly higher in farmworkers compared with non-farmworkers only in one farming period.

However, not all studies confirm that farmers have worse mental health than the general population [103]. Our review showed that 18% of the studies found farmers have a lower prevalence of mental illness than non-farmers, and 11% reported that there was no difference. For example, Otsuka and Kato [55] compared a traditional society occupation group (e.g., farmers and skilled manual workers) with an industry occupation group (e.g., managers and technicians) and revealed that the industrialized occupation group had higher levels of depression. Similarly, Liu et al. [83] found that general workers suffered from higher levels of mental stress and worse physical health compared to farmers. But, Thomas et al. [104] and Feng et al. [105] reported farmers have a lower prevalence of mental illness than the general population, although were more likely to report thinking that life was not worth living. Tomasson and Gudmundsson [106] stated that farmers were less likely to consume alcohol and that farmers’ mental health problems were 5% lower than non-farmers. Finally, Brew et al. [58] and Stain et al. [107] argued that there was no difference between mental health outcomes and wellbeing of farmers compared to non-farm workers in general, although Brew et al. [58] added that those farmers who lived more remotely had poorer mental health than non-farm workers living remotely.

### 3.4. Farm Risk Factors

Our systematic review identified several types of farmers’ risk factors. Table 3 depicts the main cited key risk factors of farmers that have been cited in the literature, namely: pesticide exposure, financial problems, climate variability/drought, physical health, isolation, role conflict, and time pressure respectively. We discuss each in more detail below.

#### 3.4.1. Pesticide Exposure

An association between pesticide exposure and farmer mental disorders has been reported in 43 reviewed studies in both developed and developing countries. As Table 3 illustrates, pesticide exposure is more cited in the developing country literature, for example studies in Brazil, India, Nepal, Philippines, Iran, Tanzania, China, Egypt, Pakistan, and Costa Rica have focused on pesticide exposure and farmer mental distress. Among the developed country literature, United States studies have also studied links between pesticide exposure and farmer mental health. Some pesticides are neurotoxic, which are said to directly affect neural systems known to cause mental illness [108,109,110,111,112,113] and depression [56,60,64,65,70,72,74,114,115,116,117,118,119,120,121,122,123]. Some studies have examined pesticide exposure in general, while others considered specific compounds such as organophosphates. Organophosphates can enter an individual’s body by the skin or through inhalation, [124] and this is associated with a range of physical symptoms (e.g dementia, Parkinson, phobia, diarrhoea, vomiting, dizziness, chest-pain, memory loss, concentration difficulties, body weakness, irritation, etc.), [60,119,123,124,125]. Wesseling et al. [60] found a relationship between acute occupational poisoning with organophosphates and psychological distress. Koh et al. [81] revealed that the association with depression was stronger amongst farmers with past pesticide poisoning episodes than amongst those with no such reported experiences. A study by Serrano-Medina et al. [126] on 140 agricultural workers with organophosphorus pesticide exposure in Mexico showed that 25% of them had major depression with suicidal attitudes, 24% had anxiety, 24% had combined depression–anxiety, and 22% of them had major depression and no psychiatric diagnosis disorder. Focus group discussions with cotton-growing farmers in India showed that during hot summer and windy seasons, some farmers reported serious health problems such as cancer, mental illness, and diabetes [127].

#### 3.4.2. Financial Pressures

Financial challenges were reported in 39 articles as negatively impacting farmers’ mental health, particularly where farming was the primary income source [8,15,19,20,31,37,79,105,128,129,130,131]. Various types of financial stress were reported by farmers in both developed and developing countries, including market prices for crops and livestock, irregular/insufficient cash flow, increased input costs, taxes, health care costs and high debt [28]. A few studies have examined the links between the 1980s farm financial crisis and mental health among U.S. farmers. During the crisis, farmers were faced with decreasing world demand, higher input costs, and low commodity prices [15,49,132,133]. Bultena et al. [133] found these factors caused farmer psychological distress, depression, lower life satisfaction, alcoholism, and even suicide. Farmers experiencing significant financial losses usually seek to make significant farm changes (e.g., through reducing the number of paid farm employees, working longer hours), diversify/change production or decide to exit [134].

Given that many other family members are impacted by farm financial problems, this has been found positively associated with farmers’ family unit stress perceptions [135]. Other mental health associations with financial stress include children numbers, broadacre production, and rental land [132]. Lawrence et al. [136] indicated that farmers are more successful in finding some alternative agronomic options for adapting to drought, but adapting to financial burdens was more difficult. There has been a positive and consistent relationship found between higher farm profit, greater well-being, and less distress amongst farmers and farm-workers [69].

#### 3.4.3. Climate Variability

Climate variability was revealed as another large risk factor for farmers in our review (25 articles of included studies focused on climate issues, mostly in developed countries). It is predicted that severe and widespread droughts will increase in the future [137]. Droughts have been categorized as slow-moving disasters which can have significant health effects, usually mediated through environmental, economic, and social pathways [76,138]. For example, 75% of farmers in a study by Walker et al. [5] reported unfavorable climate conditions and the unpredictability of the weather as their key stress in North America. Kearney et al. [28] found 60% of farmers who worked more than 40 h per week identified bad weather as ‘very stressful’ in Eastern North Carolina. In-depth interviews with 16 citrus growers in Australia, revealed that 11 of them cited drought and insufficient water allocations as potential stressors [9]. The uncertainty of weather was also reported by 70% of farmers in New York State [16]. The observed patterns of climate change have worsened farmers’ worries about the future climate and contributed to their chronic forms of mental distress [139].

Droughts have been categorized as slow-moving disasters which can have significant health effects, usually mediated through environmental, economic, and social pathways [76]. The agricultural sector is hit the hardest by drought, with farmers experiencing declined production, crop loss, and livestock failure [140]. Farmers reported a strong association between prolonged drought and stress, and higher levels of psychological morbidity [131,141,142,143,144]. Some studies reported that the major stress in a time of drought is financial hardship. For example, Edwards et al. [143] identified that drought has significant negative economic impacts, especially for farmers who reported that the drought had reduced their output substantially.

Table 3 indicates that Australia stands out in terms of the pressure of climate variability on farmer mental health in our review. Climate stressors were mentioned in 40% of Australian studies, the highest risk factor out of all possible factors. For example, Edwards et al. [145] found that the more severe the drought, the higher the adverse effects on farmer mental health. With the ongoing threat of water scarcity, falling water allocations, water reform, and drought in Australia, Wheeler et al. [26] found that some irrigators in particular industries have higher mental health problems than dryland farmers in the Murray-Darling Basin. Austin et al. [146] found that higher drought-related stress was associated with young farmers (<35 years) who live and work on a farm, and having greater financial hardship. A study by Hanigan et al. [147] showed significant associations between distress and drought duration in young rural women regardless of whether they were in farming occupations or not. However, they found that the level of drought-related distress did not differ between farmers and non-farmers in their sample.

In addition, summer heat waves are likely to have immediate effects on the prevalence and severity of farmers’ mental health. Farmers and farm workers often have no choice but to keep working, even in extreme hot weather [148]. There are also the emotional effects of landscape changes. Loss of gardens (and ‘greenness’) has been reported as a source of distress by farming families [68].

Despite several studies finding climate variability and/or drought as key risk factors on farmers, studies with a specific focus on climate variability and farmer mental health outcomes are relatively thin. Our review found that only a small amount of research (and much of it was from Australia) have focused on the effects of climate change/drought. We could find only three studies in non-developed countries (India, Ghana, and Iran) concentrating on the mental health effects of climate on farmers. Similar to our findings, a recent study by Berry et al. [149] argued that the mental health effects of climate change has received little attention in research and policy and needs greater systems thinking.

#### 3.4.4. Poor Physical Health/Past Injury

Greater mental illness amongst farmers who have poor physical health, past injury or work disability has also been found [50,52,66,150]. Farming is one of the highest risk groups for occupational injury and illness [151]. Often agricultural workers live at their worksite, so it is not surprising that an injury at work can impact their life satisfaction [54]. Distress was also related to increased physical illness on spouses and possible injury of children, which was particularly felt by farm women [152]. It has been found that farm residents with self-reported physical illness (e.g., neck, shoulder, and back pain [73]; obesity; metabolic syndrome; abdominal adiposity; and cardiovascular disease [153,154]) tend to have higher self-reported psychiatric impairment. Hawes et al. [155] found that higher body mass index (BMI) and poor sleep quality were also associated with higher depression scores. Carvalho et al. [156] found an association between work end time on the relationship between sleep onset time and farmer psychological well-being. Mazzoni et al. [157] and Stieglitz et al. [158] stated that those farmers who diagnosed with depression had a significantly higher total disability score. DeArmond and colleagues [159] found high levels of somatic symptom disorder (SSD) among farmers. SSD occurs when a person feels extreme anxiety about physical symptoms such as pain or fatigue and is significantly related to depression. Physical toxicity by agro-chemicals and damage to farmer health have been reported in a study by Kannuri and Jadhav [127]. Crandall et al. [160] argued that mental illness and the side effect of its medication can cause cognitive changes, which can put farmers at more risk of injury. Rostamabadi et al. [161] reported that musculoskeletal disorders, cuts, and fractures accounted for the most frequent injuries amongst farmer affecting their mental health. Other researchers discussed that depression and dissatisfaction with life were more strongly associated with agricultural worker injury than among other workers, and that farmers may work longer with physical health problems before receiving a disability pension than other occupations [54,100,162]. Also, as previously reported, increased symptoms of depression and suicidal thoughts were found for farm workers with a previous organophosphate poisoning [60].

#### 3.4.5. Other Risk Factors

Several other risk factors and symptoms predictive of psychological distress in farmers have been identified by researchers, such as government policies [5,17,18,22,67,77], isolation [9,17,18,37,163], heavy workload [7,37], role conflict [7,16], time pressure [7,67,79], poor housing conditions [11,22,32,66], foot and mouth disease among livestock [57,61,80,164], coal and gas development [165,166], beef crisis [167,168], lower levels of mindfulness and farmers’ work ability [78]. Overall these risk factors were stated in almost 42% of the identified studies on farmers’ mental health. Other agricultural stressors which have been identified to be common in developing countries include poor agricultural extension services/contact, poor road infrastructure, unfavourable market prices, poor access to market information, and poor access to credit facilities [36].

### 3.5. Socio-Demographic and Farm Characteristics Associated with Mental Health

We concentrate here on three of the most identified socio-demograhic and farm characteristics cited in our systematic review that have been investigated with farmer mental health, namely: gender issues (particularly for female farmers), age, and farming system type.

The literature has mainly focussed on male farmers’ mental health, even though farm women usually engage in several farm roles, which include farm labour/management, household duties and childcare [169]. Overall, our review suggested that female farmers experience more psychological distress than male farmers [14,16,21,30,50,59,62,77,80,170,171]. However, a few studies found otherwise [13,172]. Role conflict between farm and home roles, and the absence of husband support are all potential risk factors [48]. Berkowitz and Perkins [173] found that farm women who are in conflict with their husbands about farm roles, or are unhappy with their marriages, are more likely to report stress related health symptoms. Female farmers whose husbands worked more hours on the farm reported higher depressive symptoms [96]. Farm women’s depressive symptoms have also been found to be positively associated with perceived racial or ethnic discrimination and family conflict [174,175]. Alston et al. [176] found a significant increase in women’s work hours reflected their emotional distress; also that farm women are more likely to talk about their partner’s health and ignore their own. Pattnaik et al. [177] also described the feminization of agriculture as the feminization of agrarian distress.

Similar to male farmers, pesticide exposure, economic hardship and worrying about finances has often been identified as significant risk factors for female farmers’ mental health [14,16,30,67,129,174,178]. This may be a result of women undertaking additional on-farm work because of a reduction in farm paid labour [2]. Carruth and Logan [152] found that women were more likely to report depressive symptoms if they reported driving a tractor, using pesticides, and if they had a recent farm-related injury. Beseler et al. [51] found an increase in the risk of depression among women with a history of pesticide poisoning. Lu [178] examined pesticide exposure based on the duration of pesticide use amongst Philippines farmers, and reported the mean duration of pesticide exposure of 14.2 years for males and 15.4 years for females, resulting in mental and physical abnormalities in 5.4% of males and 13.3% of females. In addition, a lack of family support and listening to loud machines were also predictors of poor female mental health [14]. Alpass and collegues [67] found that farm women experienced higher levels of stress in trying to understand new farming technologies.

Age of farmers and the association with mental health issues has been discussed in-depth in the literature. Overall, younger farmers experienced higher levels of stress-related symptoms [62,85,88,179]. This was most likely associated with higher debt levels. However, Çakmur [66] found that the frequency of depressive symptoms was higher among farmers who were 35 years or older. It has also been found that there are more mental impairments observed with aging farmers [96,97,100,129,180]. Polain et al. [181] found that older farmers felt an irresistible sense of loss during prolonged drought compared with younger farmers. Scarth et al. [162] found a farmer’s depressive symptoms were not significantly related to their age. In addition, lower education levels [13,61,66,77,82,91,161,165,179], being married and having marital stress [16,82,141], and not living in a joint family [82], were associated with poorer farmer mental health.

The association with farm type (system used—such as organic farming and industry type) was also a considerable focus in the literature [182]. A study on comparing the self-reported psychological health of workers on organic and conventional horticultural farms by Cross et al. [183] showed no significant difference. However, using scores from the Short Depression Happiness Scale, organic farmers were significantly happier than conventional farmers. Similar self-reported questionnaire survey by Khan et al. [184] on 200 conventional and 157 organic farmers in Indiana, USA, found conventional farmers demonstrated a significantly higher frequency of neurological symptoms and depression problems. Similar results were found in Australian irrigation [182]. However, Brigance et al. [185] indicated that some of the risk factors that affect the mental health of organic farmers—e.g., economic insecurity, long hours of work, social isolation, and unpredictable weather conditions—are the same as the mental risk factors for conventional farmers. A recent qualitative study by Soto Mas et al. [186] on health issues in organic farming argue that although exposure to hazardous pesticides is lower amongst organic farmers, organic farming mostly relies on a few people performing a lot of tasks for cultivation, harvesting, and distribution. This issue can increase psychological and physical risk factors for organic farmers.

### 3.6. Barriers to Help-Seeking Behaviour

The final area that our systematic review covered was identifying barriers to farmers’ help-seeking behaviour. Not many of the identified studies (only 9 studies, 5% of total) reported help seeking barriers among farmers. Farmer stress and exhaustion of an individual farmer is often hidden, which may delay help-seeking behaviour [37]. Help-seeking is an active search for a relief or cure to fulfil a need and is a complex decision-making process especially for persons suffering from mental disorders [187]. Usually lack of knowledge or the belief that a person should deal with his or her mental problems alone were common reasons that decrease the possibility of individuals’ help-seeking [9,58,179]. Lack of access to mental health services in rural areas was another major burden to the delivery of appropriate mental health services [20,33,155]. Polain et al. [181] found that usually, older farmers try to access mental health support; however, practical and cultural barriers often prevented them from succeeding. Singh et al. [148] identified that existing policies were impractical and conflicts between various policies and other safety programmes were common barriers to implementation. Other barriers included farmer self-reliance, social image/stigma, negative perceptions of health professionals’ efficacy and high treatment fees [9,20]. Staniford et al. [9] and Brew et al. [58] found that farmers were half as likely to visit general practitioners or mental health professionals in the last 12 months as compared to non-farmers. Farmers often stated that it was better to manage themselves rather than access help for physical or mental health needs. Also, it has been argued that while the traditional masculine hegemony of male farmers can be a benefit to them during good times, in times of heightened stress (like drought), it can lead them to fail to address their mental health needs [188].

## 4. Discussion

This study systematically reviewed relevant research (n = 167) in order to identify the key risk factors on farming communities around the world and summarize the state of knowledge about farmer mental health. Studies reviewed were undertaken in 34 different countries, using several different assessment tools. Of the identified papers, the majority used quantitative approaches and most of them were undertaken within the past 10 years, showing increasing interest in farmers’ mental health issues, both in developed and developing countries.

Elevated levels of mental disorder within farming populations were identified by many studies e.g., [6,33,130,131,152]. However, it is also important to note that there is mixed evidence regarding the prevalence of whether mental health was worse in farmers as compared to non-farmers, but a larger portion of studies identified that psychological health disturbances were more common in farmers and farm-workers.

The most reported risk factors for farmers respectively were daily pesticides exposure [110,189], financial problems [37,129], unpredictable climate [139,143], and past injuries [52,66]. Furthermore, machinery breakdown [67], hearing loud machines [14], time pressure [79], and governmental regulations [6] were other identified risk factors. These conditions potentially make farmers more vulnerable to mental health problems. Outcomes included loss of self-esteem, withdrawal from social/community activity, relationship breakdown, hopelessness, nervousness, inability to function in occupational roles, feelings of suffocation, fatigue, insomnia, loss of control violence, and substance abuse.

The US represents the country with the highest number of farmer mental health studies, followed by Australia. American researchers were mostly focused on the associations between financial problems and farmer mental health, which has been driven by the fact that the US experienced several agricultural crises in the past few decades. Australian researchers were also concerned with financial influences impact on farmer mental health [e.g., 182]; however, Australian studies undertook the largest amount of research on climate and weather stresses for farmers, probably due to the Millennium Drought conditions in Australia in the 2000s which triggered much mental health research [188,190,191].

Most of the studies included in the systematic review used cross-sectional design (92%). The cross-sectional design prevented researchers from making strong inferences about causality and the directionality of effects reported in the studies, as the data observe the study population at only one point in time. Although several key risk factors assessed in the selected studies were significantly associated with farmer mental health status, it is unclear whether farmer stress dimensions were the primary drivers of psychological illness outcomes or not. Longitudinal research might overcome these limitations, by illustrating over the longer time, how mental distress, depression, and anxiety are connected with environmental, social and economic pressures. Also, there is a need to study the association between natural capital factors (e.g., type of farming—regenerative, organic, impact of the environment) and mental distress over the long-term, given emerging research in this space [182].

Similarly, greater consistency in assessment tools used to examine mental disorder prevalence rates among farmers may be beneficial for future research. The assessment tools used in the reviewed studies varied widely. While each of these tools may be reliable and valid indicators of clinically relevant mental disorders, they may not be directly comparable. As shown in this systematic review, farm environments (weather, environment, etc.) can significantly impact farmer health both mentally and physically. One area that will need further research in the future, is the link between climate variability, rainfall deficiency and severe drought across the world. There is a clear need for more longitudinal research in this space.

While there is extensive evidence that farming is a complex and demanding occupation with various risk factors, we suggest that access to primary care and specialist ongoing services for rural and remote communities needs greater priority. There is an argument that the impact of mental health issues for those living in rural areas is greater because many of the stresses are not paid sufficient attention, since mental health professionals are not as common in rural areas and because considerable barriers stop farmers help-seeking for mental health problems [9]. Formal help-seeking for mental health problems requires that individuals first be able to recognise that a mental health problem exists, and secondly to believe that seeking some help may be beneficial for solving their problem [192]. Limited studies to date have investigated help-seeking behaviour among farmers. Future research needs to investigate how to break down the help-seeking barriers amongst farming communities to decrease the risk of their mental disorders, as well as understanding how different types of policies can influence farmer mental health.

While this study has provided useful information to understand the issues surrounding farmer mental health, it is not without limitations. Although using systematic review principles can help researchers structure and focus literature reviews, there might be literature inadvertently missed, particularly grey literature. The issue of identifying causality of risk factors with mental health also needs careful consideration. In addition, there are many other unexamined factors which may affect farmers’ mental health but they are broader in concept and not just related to farmers. One plausible example could be solar radiation exposure for all outdoor workers, resulting in several severe adverse health effects with possible psychological consequences but also a supposed beneficial effect on some psychiatric disorders, such as depression [193]. Indeed, there might be some therapeutic potential of outdoor activities or being more outdoor vs. indoor, which might be encouraged to improve individuals’ (not specifically farmers) mental health and vitamin D status [194,195], but these aspects were not the specific focus of this study and they are left for future research.

## 5. Conclusions

The findings of this systematic review support the view that farmers’ mental health issues are a result of a complex interplay between social, environmental, and economic factors. The four most-cited risk influences on farmers’ mental health included pesticide exposure, financial difficulties, climate variabilities/drought, and poor physical health/past injuries. Studies in developed countries dominated the literature, with comparative studies suggesting that farmers generally experienced worse psychological health disturbances. Thus, future social, environmental, financial, and health policy needs to consider how best to address various mental health risk factors in the most effective way, as well as understanding how future adverse impacts from climate change can be addressed. Knowledge of risk factors affecting farmers’ mental issues is essential for reducing the burden of mental illness, hence this research is an important step in synthesising some of these important factors and outlining possible suggestions for prevention, as well as areas for future research.

## Figures and Tables

**Table 1:** Geographical locations of the selected studies.

Geographical Location	Number of Studies	Percent of Total
US	45	27%
Australia	29	17%
UK	13	8%
Mexico	10	6%
China	5	3%
South Korea	6	3%
Norway	5	3%
Iran	6	3%
India	5	3%
France	4	2%
Brazil	5	2%
Canada	3	2%
New Zealand	3	2%
Chile	2	1%
Sweden	2	1%
Turkey	2	1%
Finland	2	1%
Netherlands	2	1%
Tanzania	2	
Philippines	2	
Other countries (Iceland, Nepal, Egypt, Pakistan, United Arab Emirates, Costa Rica, Greece, Japan, Malaysia, Thailand, Bolivia, Ghana, Nigeria, Europe countries)	14	Less than 1% each

Note: Percent totals may not exactly sum to 100% due to rounding.

**Table 2:** Details of the mental health measures used in the selected studies.

Scale	Count	Percent of Total
Centre for Epidemiologic Studies-Depression (CES-D) [33]	29	18%
Questionnaires based on the Midtown Manhattan study [48]/the Warheit study [49]/the Raitasalo study [50]/the Karasek and Theorells study/asked questions such as: “has a DOCTOR ever told you that you had been diagnosed with depression requiring medication?” [51]/ “how would you rate your level of depression in the last quarter?” [52]/ “the most stressful situation you had experienced in the past month” [53]/ “have you had any injuries at work that required medical attention or treatment?” [54]/ “had any treatments or hospitalization for depression?” [55]/ “do you currently feel the defined type of stress?” [37]/ “have you had previous hospitalization for depression, by exposure to different pesticides?” [56]/21 item questionnaire [5]/Cognitive Emotion Regulation Questionnaire [57]/Copenhagen Psychosocial Questionnaire [57]/Patient Health Questionnaire (PHQ-9) [58]/Self-Reporting Questionnaire (SRQ-20) [59]	28	17%
In-depth interviews [9], focus groups [17] *	18	11%
Brief Symptom Inventory Scale [60]/15-item impact of Event Scale [61]/19 item Inventory Scale [62]/48 item Stress Scale [31]/Depression-Anxiety-Stress Scale [63]/Geriatric Depression Screening Scale [64]/Wechsler Adult Intelligence Scale [65]/Farming Family Stressor scale [7]/Beck Depression Scale (BDS) [66]/12-item Stress Scale [67]/Border Community and Immigration Stress Scale [25]/Environmental Distress Scale (EDS) [68]	16	9%
(Kessler 10) K10 [69]	12	7%
Clinical Test [70]/Medical Symptom Validity Test [71]/Mini International Neuropsychiatric Interview Diagnostic Test (MINI) [19]	12	7%
Farm Stressor Inventory [30]/Personality Assessment Inventory (PAI) [19]/Edinburgh Farming Stress Inventory (EFSI) [21]/Beck Depression Inventory (BDI) [72]/Welke’s Farm Ranch Stress Inventory [28]/Migrant Farmworker Stress Inventory [73]	11	6%
Mini-Mental State Examination (MMSE) [74]	9	5%
Hospital Anxiety and Depression (HAD) [75]	8	5%
General Health Questionnaire-12 (GHQ-12) [76]/General Health Questionnaire-28 (GHQ-28) [77]	7	4%
SF-36 [78]	4	2%
Health Option Survey (HOS) [8]/Farm Stress Survey (FSS) [79]	2	1%
Other methods (COOP/WONCA charts [80]/short-form Geriatric Depression [81]/psychological domain score of WHOQOLBREF [82]/(EQ-5D-3L) [83]/(SCL-25) [13]/(SCL-90) [84]/(ICD-9)/EuroQOL (EQ-5D)/Five-item Well-being Index (WHO-5) [85]	11	6%

Note: Percent totals may not exactly sum to 100% due to rounding. * These studies often used self-reporting methods to allow participants to tell their stories, and discuss their issues. The results of the discussions were defined as revealing farmers’ mental health issues.

**Table 3:** Key farmer mental health risk factors.

Key Risk Factors	Total Number (and %) of Studies Naming This Stress	Developed Countries (No. and %)	Developing Countries (No. and %)	USA (No. and %)	Australia (No. and %)
Pesticide exposure	43 (19%)	25 (15%)	18 (34%)	11 (16%)	1 (2%)
Finances in general (input prices/income/profit/market condition)	39 (18%)	31 (18%)	8 (18%)	14 (21%)	6 (15%)
Weather uncertainty (incl. drought and climate change)	25 (11%)	22 (13%)	3 (5%)	5 (7%)	16 (40%)
Poor physical health/past injury	23 (10%)	18 (10%)	5 (7%)	9 (13%)	1 (2%)
Farming in general/heavy workload/stress/hazards in farming	17 (8%)	12 (7%)	5 (11%)	7 (10%)	2 (5%)
Government policies and regulations/paper-work	14 (6%)	13 (8%)	1 (2%)	5 (7%)	2 (5%)
Isolation/loneliness/lack of social relationships	14 (6%)	11 (7%)	3 (7%)	4 (6%)	2 (5%)
Concern about the future of the farm/animal disease/machinery breakdown	12 (5%)	12 (7%)	0 (0%)	3 (4%)	2 (5%)
Working with family (role conflict)	12 (5%)	11 (7%)	1 (2%)	5 (7%)	2 (5%)
Time pressure	9 (4%)	7 (4%)	2 (5%)	2 (3%)	2 (5%)
Other issues—no theme identified (e.g., paddy glut/firearm exposure/media criticism/coal seam gas/electricity irrigation costs development/leaving family for work/community characteristics/work ability/lack of skilled labour/living condition/poor housing)/poor access to market information/levels of mindfulness	14 (6%)	10 (5%)	5 (9%)	4 (6%)	5 (12%)

Note: Percent totals may not exactly sum to 100% due to rounding.

## Appendix A. Risk of Bias Assessment Questions

**Selection Bias**

1. Was administered dose or exposure level adequately randomized?

2. Was allocation to study groups adequately concealed?

3. Did selection of study participants result in appropriate comparison groups?

**Confounding Bias**

4. Did the study design or analysis account for important confounding and modifying variables?

**Performance Bias**

5. Were experimental conditions identical across study groups?

6. Were the research personnel and human subjects blinded to the study group during the study?

**Attrition/Exclusion Bias**

7. Were outcome data complete without attrition or exclusion from analysis?

**Detection Bias**

8. Can we be confident in the exposure characterization?

9. Can we be confident in the outcome assessment?

**Selective Reporting Bias**

10. Were all measured outcomes reported?

**Other Sources of Bias**

11. Were there no other potential threats to internal validity (e.g., statistical methods were appropriate and researchers adhered to the study protocol)?

## References

[1] Gregoire A. (2002). The mental health of farmers. Occup. Med..

[2] Fraser C.E., Smith K.B., Judd F., Humphreys J.S., Fragar L.J., Henderson A. (2005). Farming and mental health problems and mental illness. Int. J. Soc. Psychiatry.

[3] Roy P., Tremblay G., Oliffe J.L., Jbilou J., Robertson S. (2013). Male farmers with mental health disorders: A scoping review. Aust. J. Rural Health.

[4] Price L., Evans N. (2009). From stress to distress: Conceptualizing the British family farming patriarchal way of life. J. Rural Stud..

[5] Walker J.L., Walker L.S., MacLennan P.M. (1986). An informal look at farm stress. Psychol. Rep..

[6] Raine G. (1999). Causes and effects of stress on farmers: A qualitative study. Health Educ. J..

[7] McShane C.J., Quirk F., Swinbourne A. (2016). Development and validation of a work stressor scale for Australian farming families. Aust. J. Rural Health.

[8] Vega W., Warheit G., Palacio R. (1985). Psychiatric symptomatology among Mexican American farmworkers. Soc. Sci. Med..

[9] Staniford A.K., Dollard M.F., Guerin B. (2009). Stress and help-seeking for drought-stricken citrus growers in the Riverland of South Australia. Aust. J. Rural Health.

[10] Firth H.M., Williams S.M., Herbison G.P., McGee R.O. (2007). Stress in New Zealand farmers. Stress Health J. Int. Soc. Investig. Stress.

[11] Mora D.C., Quandt S.A., Chen H., Arcury T.A. (2016). Associations of Poor Housing with Mental Health Among North Carolina Latino Migrant Farmworkers. J. Agromedicine.

[12] Hovey J.D., Magaña C.G. (2002). Exploring the Mental Health of Mexican Migrant Farm Workers in the Midwest: Psychosocial Predictors of Psychological Distress and Suggestions for Prevention and Treatment. J. Psychol..

[13] Logstein B. (2016). Farm-Related Concerns and Mental Health Status Among Norwegian Farmers. J. Agromedicine.

[14] Hanklang S., Kaewboonchoo O., Morioka I., Plernpit S.-A. (2016). Gender Differences in Depression Symptoms Among Rice Farmers in Thailand. Asia Pac. J. Public Health.

[15] Williams R.T. (1996). The on-going farm crisis: Extension leadership in rural communities. J. Ext..

[16] Hedlund D., Berkowitz A. (1979). The incidence of socialpsychologicl stress in farm families. Int. J. Sociol. Fam..

[17] Hossain D., Eley R., Coutts J., Gorman D. (2008). Mental health of farmers in Southern Queensland: Issues and support. Aust. J. Rural Health.

[18] Hiott A.E., Grzywacz J.G., Davis S.W., Quandt S.A., Arcury T.A. (2008). Migrant Farmworker Stress: Mental Health Implications. J. Rural Health.

[19] Hovey J.D., Magaña C.G. (2002). Psychosocial Predictors of Anxiety Among Immigrant Mexican Migrant Farmworkers: Implications for Prevention and Treatment. Cult. Divers. Ethn. Minority Psychol..

[20] Ramos A., Su D., Lander L., Rivera R. (2015). Stress Factors Contributing to Depression Among Latino Migrant Farmworkers in Nebraska. J. Immigr. Minority Health.

[21] Deary I.J., Willock J., McGregor M. (1997). Stress In Farming. Stress Med..

[22] Grzywacz J.G., Quandt S.A., Chen H., Isom S., Kiang L., Vallejos Q., Arcury T.A. (2010). Depressive Symptoms Among Latino Farmworkers Across the Agricultural Season: Structural and Situational Influences. Cult. Divers. Ethn. Minority Psychol..

[23] Bin H., Lamm F., Tipples R. (2008). The Impact of Stressors on the Psychological Wellbeing of New Zealand Farmers and The Development of an Explanatory Conceptual Model. Policy Pract. Health Saf..

[24] Simkin S., Hawton K., Fagg J., Malmberg A. (1998). Stress in farmers: A survey of farmers in England and Wales. Occup. Environ. Med..

[25] Carvajal S., Kibor C., McClelland D., Ingram M., Zapien J., Torres E., Redondo F., Rodriguez K., Rubio-Goldsmith R., Meister J. (2014). Stress and Sociocultural Factors Related to Health Status Among US–Mexico Border Farmworkers. J. Immigr. Minority Health.

[26] Wheeler S.A., Zuo A., Loch A. (2018). Water torture: Unravelling the psychological distress of irrigators in Australia. J. Rural Stud..

[27] Lunner Kolstrup C., Kallioniemi M., Lundqvist P., Kymäläinen H.-R., Stallones L., Brumby S. (2013). International Perspectives on Psychosocial Working Conditions, Mental Health, and Stress of Dairy Farm Operators. J. Agromedicine.

[28] Kearney G.D., Rafferty A.P., Hendricks L.R., Allen D.L., Tutor-Marcom R. (2014). A cross-sectional study of stressors among farmers in eastern North Carolina. N. C. Med. J..

[29] Williams R. (2001). The ongoing farm crisis: Health, mental health and safety issues in Wisconsin. Rural Ment. Health.

[30] Walker L.S., Walker J.L. (1987). Stressors and symptoms predictive of distress in farmers. Fam. Relat..

[31] Simsek Z., Ersin F., Kirmizitoprak E. (2015). Development of the Seasonal Migrant Agricultural Worker Stress Scale in Sanliurfa, Southeast Turkey. J. Agromedicine.

[32] O’Connor K., Stoecklin-Marois M., Schenker M.B. (2015). Examining nervios among immigrant male farmworkers in the MICASA study: Sociodemographics, housing conditions and psychosocial factors. J. Immigr. Minority Health.

[33] Terrazas S.R., McCormick A. (2018). Coping Strategies That Mitigate Against Symptoms of Depression Among Latino Farmworkers. Hisp. J. Behav. Sci..

[34] Liu B.-P., Qin P., Liu Y.-Y., Yuan L., Gu L.-X., Jia C.-X. (2018). Mental disorders and suicide attempt in rural China. Psychiatry Res..

[35] Glasscock D.J., Rasmussen K., Carstensen O., Hansen O.N. (2006). Psychosocial factors and safety behaviour as predictors of accidental work injuries in farming. Work Stress.

[36] Olowogbon T.S., Yoder A.M., Fakayode S.B., Falola A.O. (2019). Agricultural stressors: Identification, causes and perceived effects among Nigerian crop farmers. J. Agromedicine.

[37] Kallioniemi M.K., Simola A., Kaseva J., Kymäläinen H.-R. (2016). Stress and Burnout Among Finnish Dairy Farmers. J. Agromedicine.

[38] Page A.N., Fragar L.J. (2002). Suicide in Australian farming. Aust. N. Z. Psychiatry.

[39] Das A. (2011). Farmers’ Suicide in India: Implications for Public Mental Health. Int. J. Soc. Psychiatry.

[40] Kunde L., Kõlves K., Kelly B., Reddy P., De Leo D. (2017). Pathways to suicide in Australian farmers: A life chart analysis. Int. J. Environ. Res. Public Health.

[41] Perceval M., Kõlves K., Reddy P., De Leo D. (2017). Farmer suicides: A qualitative study from Australia. Occup. Med..

[42] World Health Organization (2007). Mental Health: Strengthening Mental Health Promotion.

[43] World Health Organization (2007). Depression and Other Common Mental Disorders: Global Health Estimates.

[44] World Health Organization (2007). Promoting Mental Health: Concepts, Emerging Evidence, Practice: Summary Report.

[45] Department of Health and Ageing National Mental Health Report 2013: Tracking Progress of Mental Health Reform in Australia 1993–2011.

[46] Moher D., Liberati A., Tetzlaff J., Altman D.G. (2009). Preferred reporting items for systematic reviews and meta-analyses: The PRISMA statement. Ann. Intern. Med..

[47] Handbook for Conducting a Literature-Based Health Assessment Using OHAT Approach for Systematic Review and Evidence Integration.

[48] Berkowitz A., Wesley Perkins H. (1984). Stress among farm women: Work and family as interacting systems. J. Marriage Fam..

[49] Ortega S.T., Johnson D.R., Beeson P.G., Craft B.J. (1994). The farm crisis and mental health: A longitudinal study of the 1980s. Rural Sociol..

[50] Kallioniemi M.K., Simola A.J.K., Kymäläinen H.-R., Vesala H.T., Louhelainen J.K. (2009). Mental symptoms among Finnish farm entrepreneurs. Ann. Agric. Environ. Med..

[51] Beseler C., Stallones L., Hoppin J.A., Alavanja M.C., Blair A., Keefe T., Kamel F. (2006). Depression and pesticide exposures in female spouses of licensed pesticide applicators in the agricultural health study cohort. J. Occup. Environ. Med..

[52] Onwuameze O.E., Paradiso S., Peek-Asa C., Donham K.J., Rautiainen R.H. (2013). Modifiable risk factors for depressed mood among farmers. Ann. Clin. Psychiatry.

[53] Fennell K.M., Jarrett C.E., Kettler L.J., Dollman J., Turnbull D.A. (2016). “Watching the bank balance build up then blow away and the rain clouds do the same”: A thematic analysis of South Australian farmers’ sources of stress during drought. J. Rural Stud..

[54] Zwerling C., Sprince N., Wallace R., Davis C., Whitten P., Heeringa S. (1995). Occupational injuries among agricultural workers 51 to 61 years old: A national study. J. Agric. Saf. Health.

[55] Otsuka K., Kato S. (2000). Relationship between Diagnostic Subtypes of Depression and Occupation in Japan. Psychopathology.

[56] Weisskopf M.G., Moisan F., Tzourio C., Rathouz P.J., Elbaz A. (2013). Pesticide Exposure and Depression Among Agricultural Workers in France. Am. J. Epidemiol..

[57] Garnefski N., Baan N., Kraaij V. (2005). Psychological distress and cognitive emotion regulation strategies among farmers who fell victim to the foot-and-mouth crisis. Personal. Individ. Differ..

[58] Brew B., Inder K., Allen J., Thomas M., Kelly B. (2016). The health and wellbeing of Australian farmers: A longitudinal cohort study. BMC Public Health.

[59] Poletto Â.R., Gontijo L.A. (2012). Family farming workers mental health in a microrregion in southern Brazil. Work.

[60] Wesseling C., van Wendel de Joode B., Keifer M., London L., Mergler D., Stallones L. (2010). Symptoms of psychological distress and suicidal ideation among banana workers with a history of poisoning by organophosphate or n-methyl carbamate pesticides. Occup. Environ. Med..

[61] Olff M., Koeter M.W.J., Van Haaften E.H., Kersten P.H., Gersons B.P.R. (2005). Impact of a foot and mouth disease crisis on post-traumatic stress symptoms in farmers. Br. J. Psychiatry.

[62] Walker J.L., Walker L.J.S. (1988). Self-reported stress symptoms in farmers. J. Clin. Psychol..

[63] McShane C.J., Quirk F. (2009). Mediating and moderating effects of work–home interference upon farm stresses and psychological distress. Aust. J. Rural Health.

[64] Kim J., Ko Y., Lee W.J. (2013). Depressive symptoms and severity of acute occupational pesticide poisoning among male farmers. Occup. Environ. Med..

[65] Hong Z.-R., Hong S.-Y., Han M.-J., Lee H.-S., Gil H.-O., Yang J.-O., Lee E.-Y., Hong S.-Y. (2008). Clinical observation of 12 farmers who believe themselves to have suffered from chronic pesticide intoxication. Korean J. Intern. Med..

[66] Çakmur H. (2014). Health Risks Faced by Turkish Agricultural Workers. Sci. World J..

[67] Alpass F., Flett R., Humphries S., Massey C., Morriss S., Long N. (2004). Stress in Dairy Farming and the Adoption of New Technology. Int. J. Stress Manag..

[68] Albrecht G., Sartore G.-M., Connor L., Higginbotham N., Freeman S., Kelly B., Stain H., Tonna A., Pollard G. (2007). Solastalgia: The Distress Caused by Environmental Change. Aust. N. Z. Psychiatry.

[69] Peel D., Berry H.L., Schirmer J. (2015). Perceived profitability and well-being in Australian dryland farmers and irrigators. Aust. J. Rural Health.

[70] Atreya K., Kumar Sitaula B., Overgaard H., Man Bajracharya R., Sharma S. (2012). Knowledge, attitude and practices of pesticide use and acetylcholinesterase depression among farm workers in Nepal. Int. J. Environ. Health Res..

[71] Mackenzie Ross S.J., Brewin C.R., Curran H.V., Furlong C.E., Abraham-Smith K.M., Harrison V. (2010). Neuropsychological and psychiatric functioning in sheep farmers exposed to low levels of organophosphate pesticides. Neurotoxicol. Teratol..

[72] Hong S.-Y., Jeong D.-S., Gil H.-W., Yang J.-O., Lee E.-Y., Hong S.-Y. (2009). The estimation of pesticide exposure in depression scores: In case of Korean orchard farmers. J. Pest Sci..

[73] Tribble A.G., Summers P., Chen H., Quandt S.A., Arcury T.A. (2016). Musculoskeletal pain, depression, and stress among Latino manual laborers in North Carolina. Arch. Environ. Occup. Health.

[74] Bayrami M., Hashemi T., Malekirad A.A., Ashayeri H., Faraji F., Abdollahi M. (2012). Electroencephalogram, cognitive state, psychological disorders, clinical symptom, and oxidative stress in horticulture farmers exposed to organophosphate pesticides. Toxicol. Ind. Health.

[75] Guillien A., Laurent L., Soumagne T., Puyraveau M., Laplante J.-J., Andujar P., Annesi-Maesano I., Roche N., Degano B., Dalphin J.-C. (2018). Anxiety and depression among dairy farmers: The impact of COPD. Int. J. Chron. Obs. Pulm. Dis..

[76] King D., Lane A., MacDougall C., Greenhill J. (2009). The Resilience and Mental Health and Wellbeing of Farm Families Experiencing Climate Variation in South Australia.

[77] Booth N.J., Lloyd K. (2000). Stress in farmers. Int. J. Soc. Psychiatry.

[78] Rostamabadi A., Mazloumi A., Rahimi Foroushani A. (2014). Work Ability Index (WAI) and its health-related determinants among Iranian farmers working in small farm enterprises. J. Occup. Health.

[79] Eberhardt B.J., Pooyan A. (1990). Development of the Farm Stress Survey: Factorial Structure, Reliability, and Validity. Educ. Psychol. Meas..

[80] Hannay D., Jones R. (2002). The effects of foot-and-mouth on the health of those involved in farming and tourism in Dumfries and Galloway. Eur. J. Gen. Pract..

[81] Koh S.-B., Kim T.H., Min S., Lee K., Kang D.R., Choi J.R. (2017). Exposure to pesticide as a risk factor for depression: A population-based longitudinal study in Korea. Neurotoxicology.

[82] Saxena S., Rp V., Soman B. (2013). Exposure to firearm: Impact on psychological health in central India. Indian J. Community Health.

[83] Liu X., Gu S., Duan S., Wu Y., Ye C., Wang J., Dong H. (2017). Comparative Study on Health-Related Quality of Life of Farmers and Workers. Value Health Reg. Issues.

[84] Wang L. (2005). Stress and mental health of farmer-workers. Chin. J. Ind. Hyg. Occup. Dis..

[85] Jessie G., Deborah De M., Mathijn W., Christophe V. (2018). What’s up with the self-employed? A cross-national perspective on the self-employed’s work-related mental well-being. SSM Popul. Health.

[86] Stallones L., Beseler C. (2004). Safety practices and depression among farm residents. Ann. Epidemiol..

[87] Syson-Nibbs L., Saul C., Cox P. (2006). Tideswell health survey: A population survey of the health needs and service utilization of a farming community. Public Health.

[88] Elliott M., Heaney C.A., Wilkins J.R., Mitchell G.L., Bean T. (1995). Depression and Perceived Stress Among Cash Grain Farmers in Ohio. J. Agric. Saf. Health.

[89] Bean T.L., Nolan J.A. (2008). Recognize and manage the stress of farm life. Agriculture National Resources.

[90] Kolstrup C., Lundqvist P., Pinzke S. (2008). Psychosocial Work Environment Among Employed Swedish Dairy and Pig Farmworkers. J. Agromedicine.

[91] Sanne B., Mykletun A., Moen B.E., Dahl A.A., Tell G.S. (2004). Farmers are at risk for anxiety and depression: The Hordaland Health Study. Occup. Med..

[92] Wallis A., Dollard M.F. (2008). Local and global factors in work stress—the Australian dairy farming examplar. Scand. J. Work Environ. Health.

[93] Arcury T.A., Sandberg J.C., Talton J.W., Laurienti P.J., Daniel S.S., Quandt S.A. (2018). Mental Health Among Latina Farmworkers and Other Employed Latinas in North Carolina. J. Rural Ment. Health.

[94] Cohidon C., Santin G., Imbernon E., Goldberg M. (2010). Working conditions and depressive symptoms in the 2003 decennial health survey: The role of the occupational category. Soc. Psychiatry Psychiatr. Epidemiol..

[95] Hounsome B., Edwards R., Hounsome N., Edwards-Jones G. (2012). Psychological Morbidity of Farmers and Non-farming Population: Results from a UK Survey. Community Ment. Health J..

[96] Konstantinos D., Eleni S., Eleni J., Nikolaos C., John E., Michalis L. (2013). Does Farming Have an Effect on Health Status? A Comparison Study in West Greece. Int. J. Environ. Res. Public Health.

[97] Rayens M.K., Reed D.B. (2014). Predictors of Depressive Symptoms in Older Rural Couples: The Impact of Work, Stress and Health. J. Rural Health.

[98] Thelin A.G. (1998). Working environment conditions in rural areas according to psychosocial indices. Ann. Agric. Environ. Med..

[99] Torske M.O., Hilt B., Glasscock D., Lundqvist P., Krokstad S. (2015). Anxiety and depression symptoms among farmers. The HUNT Study, Norway. J. Agromedicine.

[100] Torske M.O., Hilt B., Bjørngaard J.H., Glasscock D., Krokstad S. (2015). Disability pension and symptoms of anxiety and depression: A prospective comparison of farmers and other occupational groups. The HUNT Study, Norway. BMJ Open.

[101] Yin H., Xu G., Tian H., Yang G., Wardenaar K.J., Schoevers R.A. (2018). The prevalence, age-of-onset and the correlates of DSM-IV psychiatric disorders in the Tianjin Mental Health Survey (TJMHS). Psychol. Med..

[102] Ulrich A., Molina Y., Briant K.J., Onstad L.E., Copeland W., Holte S.E., Thompson B. (2018). Stress Among Latinos: Does it Vary by Occupation and Agricultural Season?. J. Occup. Environ. Med..

[103] Judd F., Jackson H., Fraser C., Murray G., Robins G., Komiti A. (2006). Understanding suicide in Australian farmers. Soc. Psychiatry Psychiatr. Epidemiol..

[104] Thomas H.V., Lewis G., Thomas D.R., Salmon R.L., Chalmers R.M., Coleman T.J., Kench S.M., Morgan-Capner P., Meadows D., Sillis M. (2003). Mental health of British farmers. Occup. Environ. Med..

[105] Feng D., Ji L., Xu L. (2015). Effect of subjective economic status on psychological distress among farmers and non-farmers of rural China. Aust. J. Rural Health.

[106] Tomasson K., Gudmundsson G. (2009). Mental health and wellbeing in Icelandic farmers. Laeknabladid.

[107] Stain H., Kelly B., Lewin T., Higginbotham N., Beard J., Hourihan F. (2008). Social networks and mental health among a farming population. Soc. Psychiatry Psychiatr. Epidemiol..

[108] Malekirad A.A., Faghih M., Mirabdollahi M., Kiani M., Fathi A., Abdollahi M. (2013). Neurocognitive, mental health, and glucose disorders in farmers exposed to Organophosphorus pesticides.(Report). Arch. Hyg. Toksikol..

[109] Jamal G.A., Hansen S., Pilkington A., Buchanan D., Gillham R.A., Abdel-Azis M., Julu P.O.O., Al-Rawas S.F., Hurley F., Ballantyne J.P. (2002). A clinical neurological, neurophysiological, and neuropsychological study of sheep farmers and dippers exposed to organophosphate pesticides. Occup. Environ. Med..

[110] Aiwerasia V.F.N., David N.M., Timo J.P., Michael P.S., Godson M. (2001). Acute health effects of organophosphorus pesticides on Tanzanian small-scale coffee growers. J. Exp. Anal. Environ. Epidemiol..

[111] Steenland K., Jenkins B., Ames R.G., O’Malley M., Chrislip D., Russo J. (1994). Chronic neurological sequelae to organophosphate pesticide poisoning. Am. J. Public Health.

[112] Corral S.A., de Angel V., Salas N., Zúñiga-Venegas L., Gaspar P.A., Pancetti F. (2017). Cognitive impairment in agricultural workers and nearby residents exposed to pesticides in the Coquimbo Region of Chile. Neurotoxicol. Teratol..

[113] Muñoz-Quezada M.T., Lucero B., Iglesias V., Levy K., Muñoz M.P., Achú E., Cornejo C., Concha C., Brito A.M., Villalobos M. (2017). Exposure to organophosphate (OP) pesticides and health conditions in agricultural and non-agricultural workers from Maule, Chile. Int. J. Environ. Health Res..

[114] Harrison V., Mackenzie Ross S. (2016). Anxiety and depression following cumulative low-level exposure to organophosphate pesticides. Environ. Res..

[115] Quandt S.A., Chen H., Grzywacz J.G., Vallejos Q.M., Galvan L., Arcury T.A. (2010). Cholinesterase depression and its association with pesticide exposure across the agricultural season among Latino farmworkers in North Carolina. Environ. Health Perspect..

[116] Beseler C. (2005). Diagnosed Depression and Low, Intermediate, and High Pesticide Exposures in Iowa and North Carolina Farm Applicators and Their Spouses Enrolled in the Agricultural Health Study. Ph.D. Thesis.

[117] Da Silva V.d.S.P., de Mello M.S.C., Otero U.B. (2016). Exposure to pesticides and mental disorders in a rural population of Southern Brazil. Neurotoxicology.

[118] Siegel M., Starks S., Sanderson W., Kamel F., Hoppin J., Gerr F. (2017). Organic solvent exposure and depressive symptoms among licensed pesticide applicators in the Agricultural Health Study. Int. Arch. Occup. Environ. Health.

[119] Povey A.C., McNamee R., Alhamwi H., Stocks S.J., Watkins G., Burns A., Agius R. (2014). Pesticide exposure and screen-positive neuropsychiatric disease in British sheep farmers. Environ. Res..

[120] Zhang X., Wu M., Yao H., Yang Y., Cui M., Tu Z., Stallones L., Xiang H. (2016). Pesticide poisoning and neurobehavioral function among farm workers in Jiangsu, People’s Republic of China. Cortex.

[121] Fariba T., Gholamhassan V., Mohammad A., Ali Akbar M. (2016). A Comparative Study of the Quality of Life, Depression, Anxiety and Stress in Farmers Exposed to Organophosphate Pesticides with those in a Control Group. J. Chem. Health Risks.

[122] Conti C.L., Barbosa W.M., Simão J.B.P., Álvares-da-Silva A.M. (2018). Pesticide exposure, tobacco use, poor self-perceived health and presence of chronic disease are determinants of depressive symptoms among coffee growers from Southeast Brazil. Psychiatry Res..

[123] Suten Geofrey M., Ezra Jonathan M., Aiwerasia Vera N., Simon M. (2018). Health Symptoms Associated with Pesticides Exposure among Flower and Onion Pesticide Applicators in Arusha Region. Ann. Glob. Health.

[124] Mearns J., Dunn J., Lees-Haley P.R. (1994). Psychological effects of organophosphate pesticides: A review and call for research by psychologists. J. Clin. Psychol..

[125] Beshwari M.M.M., Bener A., Ameen A., Al-Mehdi A.M., Ouda H.Z., Pasha M.A.H. (1999). Pesticide-related health problems and diseases among farmers in the United Arab Emirates. Int. J. Environ. Health Res..

[126] Serrano-Medina A., Ugalde-Lizárraga A., Bojorquez-Cuevas M.S., Garnica-Ruiz J., González-Corral M.A., García-Ledezma A., Pineda-García G., Cornejo-Bravo J.M. (2019). Neuropsychiatric Disorders in Farmers Associated with Organophosphorus Pesticide Exposure in a Rural Village of Northwest México. Int. J. Environ. Res. Public Health.

[127] Kannuri N.K., Jadhav S. (2018). Generating toxic landscapes: Impact on well-being of cotton farmers in Telangana, India. Anthropol. Med..

[128] Bryant L., Garnham B. (2014). Economies, ethics and emotions: Farmer distress within the moral economy of agribusiness. J. Rural Stud..

[129] Pulgar C., Trejo G., Suerken C., Ip E., Arcury T., Quandt S. (2016). Economic Hardship and Depression Among Women in Latino Farmworker Families. J. Immigr. Minority Health.

[130] Truchot D., Andela M. (2018). Burnout and hopelessness among farmers: The Farmers Stressors Inventory. Soc. Psychiatry Psychiatr. Epidemiol..

[131] Kureshi J.S., Somsundaram K. (2018). Assessment of occupational stress among farmers in Aurangabad district, Maharashtra. Int. J. Community Med. Public Health.

[132] Belyea M.J., Lobao L.M. (1990). Psychosocial Consequences of Agricultural Transformation: The Farm Crisis and Depression. Rural Sociol..

[133] Bultena G., Lasley P., Geller J. (1986). The farm crisis: Patterns and impacts of financial distress among Iowa farm families. Rural Sociol..

[134] Bryant L., Garnham B. (2013). Beyond discourses of drought: The micro-politics of the wine industry and farmer distress. J. Rural Stud..

[135] Welke C. (2004). Farm/Ranch Stressors and the Distress and Job Satisfaction of Farm Family Members: The Buffering Effects of Perceived Social Support. Ph.D. Thesis.

[136] Lawrence P., Maxwell B., Rew L., Ellis C., Bekkerman A. (2018). Vulnerability of dryland agricultural regimes to economic and climatic change. Ecol. Soc..

[137] Dai A. (2013). Increasing drought under global warming in observations and models. Nat. Clim. Chang..

[138] Vins H., Bell J., Saha S., Hess J.J. (2015). The mental health outcomes of drought: A systematic review and causal process diagram. Int. J. Environ. Res. Public Health.

[139] Ellis N.R., Albrecht G.A. (2017). Climate change threats to family farmers’ sense of place and mental wellbeing: A case study from the Western Australian Wheatbelt. Soc. Sci. Med..

[140] Berry H.L., Kelly B.J., Hanigan I.C., Coates J.H., McMichael A.J., Welsh J.A., Kjellstrom T. (2008). Rural mental health impacts of climate change. Commissioned Report for the Garnaut Climate Change Review.

[141] Stain H.J., Kelly B., Carr V.J., Lewin T.J., Fitzgerald M., Fragar L. (2011). The psychological impact of chronic environmental adversity: Responding to prolonged drought. Soc. Sci. Med..

[142] Sartore G.-M., Kelly B., Stain H.J. (2007). Drought and its effect on mental health: How GPs can help. Aust. Fam. Physician.

[143] Edwards B., Gray M., Hunter B. (2009). A sunburnt country: The economic and financial impact of drought on rural and regional families in Australia in an era of climate change. Aust. J. Lab. Econ..

[144] Acharibasam J.W., Anuga S.W. (2018). Psychological distance of climate change and mental health risks assessment of smallholder farmers in Northern Ghana: Is habituation a threat to climate change?. Clim. Risk Manag..

[145] Edwards B., Gray M., Hunter B. (2015). The impact of drought on mental health in rural and regional Australia. Soc. Indic. Res..

[146] Austin E.K., Handley T., Kiem A.S., Rich J.L., Lewin T.J., Askland H.H., Askarimarnani S.S., Perkins D.A., Kelly B.J. (2018). Drought-related stress among farmers: Findings from the Australian Rural Mental Health Study. Med. J. Aust..

[147] Hanigan I.C., Schirmer J., Niyonsenga T. (2018). Drought and Distress in Southeastern Australia. EcoHealth.

[148] Singh S., Hanna E.G., Kjellstrom T. (2013). Working in Australia’s heat: Health promotion concerns for health and productivity. Health Promot. Int..

[149] Berry H.L., Waite T.D., Dear K.B., Capon A.G., Murray V. (2018). The case for systems thinking about climate change and mental health. Nat. Clim. Chang..

[150] Linn J.G., Husaini B.A. (1987). Determinants of psychological depression and coping behaviors of Tennessee farm residents. J. Community Psychol..

[151] Dixon J., Welch N. (2000). Researching the Rural–Metropolitan Health Differential Using the ‘Social Determinants of Health’. Aust. J. Rural Health.

[152] Carruth A., Logan C. (2002). Depressive Symptoms in Farm Women: Effects of Health Status and Farming Lifestyle Characteristics, Behaviors, and Beliefs. Publ. Health Promot. Dis. Prev..

[153] Brumby S., Chandrasekara A., McCoombe S., Kremer P., Lewandowski P. (2012). Cardiovascular risk factors and psychological distress in Australian farming communities. Aust. J. Rural Health.

[154] Schulz P.S., Zimmerman L., Johansson P. (2018). Seasonal Work and Cardiovascular Risk Factors in Farmers. J. Cardiovasc. Nurs..

[155] Hawes N.J., Wiggins A.T., Reed D.B., Hardin-Fanning F. (2019). Poor sleep quality is associated with obesity and depression in farmers. Public Health Nurs..

[156] Carvalho F.G., de Souza C.M., Hidalgo M.P.L. (2018). Work routines moderate the association between eveningness and poor psychological well-being. PLoS ONE.

[157] Mazzoni S.E., Boiko P.E., Katon W.J., Russo J. (2007). Depression and disability in seasonal and migrant Hispanic agricultural workers. Gen. Hosp. Psychiatry.

[158] Stieglitz J., Schniter E., von Rueden C., Kaplan H., Gurven M. (2015). Functional Disability and Social Conflict Increase Risk of Depression in Older Adulthood Among Bolivian Forager-Farmers. J. Gerontol. Ser. B Psychol. Sci. Soc. Sci..

[159] DeArmond S., Stallones L., Chen P., Sintek E. (2006). Depression and somatic symptoms within the farming community. J. Agric. Saf. Health.

[160] Crandall C.S., Fullerton L., Olson L., Sklar D.P., Zumwalt R. (1997). Farm-related injury mortality in New Mexico, 1980–1991. Accid. Anal. Prev..

[161] Rostamabadi A., Jahangiri M., Naderi Mansourabadi B., Javid M., Ghorbani M., Banaee S. (2019). Prevalence of chronic diseases and occupational injuries and their influence on the health-related quality of life among farmers working in small-farm enterprises. J. Agromedicine.

[162] Scarth R.D., Zwerling C., Lewis M.Q., Burmeister L.F. (1997). Depression and risk factors among Iowa farmers. J. Agromedicine.

[163] Logstein B. (2016). Predictors of mental complaints among Norwegian male farmers. Occup. Med..

[164] Peck D.F., Grant S., McArthur W., Godden D. (2002). Psychological impact of foot-and-mouth disease on farmers. J. Ment. Health.

[165] Huth N., Cocks B., Dalgliesh N., Poulton P., Marinoni O., Garcia J. (2018). Farmers’ perceptions of coexistence between agriculture and a large scale coal seam gas development. Agric. Hum. Values.

[166] Morgan M.I., Hine D.W., Bhullar N., Dunstan D.A., Bartik W. (2016). Fracked: Coal seam gas extraction and farmers’ mental health. J. Environ. Psychol..

[167] Eisner C.S., Neal R.D., Scaife B. (1999). The effect of the 1996 ‘beef crisis’ on depression and anxiety in farmers and non-farming controls. Br. J. Psychiatry.

[168] Eddy P., Wertheim E.H., Hale M.W., Wright B.J. (2019). Trait Mindfulness Helps Explain the Relationships Between Job Stress, Physiological Reactivity, and Self-Perceived Health. J. Occup. Environ. Med..

[169] Mulder P.L., Kenkel M., Shellenberger S., Constantine M., Streiegel R., Sears S., Jumper-Thurman P., Kalodner M., Danda C., Hager A. (2000). The Behavioral Health Care Needs of Rural Women.

[170] Lee H., Cho S.-Y., Kim J.-S., Yoon S.-Y., Kim B.-I., An J.-M., Kim K.-B. (2019). Difference in health status of Korean farmers according to gender. Ann. Occup. Environ. Med..

[171] Weigel R.R., Weigel D.J. (1987). Identifying stressors and coping strategies in two-generation farm families. Fam. Relat..

[172] Gunn K. (2008). Farmers’ Stress and Coping in a Time of Drought. Ph.D. Thesis.

[173] Berkowitz A.D., Perkins H.W. (1985). Correlates of Psychosomatic Stress Symptoms among Farm Women: A Research Note on Farm and Family Functioning. J. Hum. Stress.

[174] Zapata Roblyer M.I., Grzywacz J.G., Suerken C.K., Trejo G., Ip E.H., Arcury T.A., Quandt S.A. (2015). Interpersonal and social correlates of depressive symptoms among Latinas in farmworker families living in North Carolina. Women Health.

[175] Millondaga K.J. (2018). Mothers, wives, and farmers: Stories of women ‘gone mad’. Asian J. Women’s Stud..

[176] Alston M., Clarke J., Whittenbury K. (2018). Contemporary Feminist Analysis of Australian Farm Women in the Context of Climate Changes. Soc. Sci..

[177] Pattnaik I., Lahiri-Dutt K., Lockie S., Pritchard B. (2018). The feminization of agriculture or the feminization of agrarian distress? Tracking the trajectory of women in agriculture in India. Asia Pac. Econ..

[178] Lu J.L. (2017). Assessment of Pesticide-Related Pollution and Occupational Health of Vegetable Farmers in Benguet Province, Philippines. J. Health Pollut..

[179] Wang D., Ma J., Tan L., Chen Y., Li X., Tian X., Zhou X., Liu X. (2017). Epidemiology of severe mental illness in Hunan province in central China during 2014-2015: A multistage cross-sectional study. PLoS ONE.

[180] McClure H., Josh Snodgrass J., Martinez C., Squires E., Jiménez R., Isiordia L., Eddy J., McDade T., Small J. (2015). Stress, Place, and Allostatic Load Among Mexican Immigrant Farmworkers in Oregon. J. Immigr. Minority Health.

[181] Polain J.D., Berry H.L., Hoskin J.O. (2011). Rapid change, climate adversity and the next ‘big dry’: Older farmers’ mental health. Aust. J. Rural Health.

[182] Daghagh Yazd S., Wheeler S.A., Zuo A. (2019). Exploring the Drivers of Irrigator Mental Health in the Murray–Darling Basin, Australia. Sustainability.

[183] Cross P., Edwards R.T., Hounsome B., Edwards-Jones G. (2008). Comparative assessment of migrant farm worker health in conventional and organic horticultural systems in the United Kingdom. Sci. Total Environ..

[184] Khan K.M., Baidya R., Aryal A., Farmer J.R., Valliant J. (2018). Neurological and mental health outcomes among conventional and organic farmers in Indiana, USA. Ann. Agric. Environ. Med..

[185] Brigance C., Soto Mas F., Sanchez V., Handal A.J. (2018). The Mental Health of the Organic Farmer: Psychosocial and Contextual Actors. Workplace Health Saf..

[186] Soto Mas F., Handal A.J., Rohrer R.E., Tomalá Viteri E. (2018). Health and safety in organic farming: A qualitative study. J. Agromedicine.

[187] Cornally N., McCarthy G. (2011). Help-seeking behaviour: A concept analysis. Int. J. Nurs. Pract..

[188] Alston M., Kent J. (2008). The Big Dry: The link between rural masculinities and poor health outcomes for farming men. J. Sociol..

[189] Beseler C.L., Stallones L. (2008). A Cohort Study of Pesticide Poisoning and Depression in Colorado Farm Residents. Ann. Epidemiol..

[190] Wheeler S.A., Zuo A., Xu Y., Grafton Q., Daghagh Yazd S. (2019). Emergency Drought Relief Package—Health and Resilience Services: An Evidence Check.

[191] Daghagh Yazd S., Wheeler S.A., Zuo A. (2020). Understanding the impacts of water scarcity and socio-economic demographics on farmer mental health in the Murray-Darling Basin. Ecol. Econ..

[192] Henderson C., Evans-Lacko S., Thornicroft G. (2013). Mental illness stigma, help seeking, and public health programs. Am. J. Public Health.

[193] Modenese A., Korpinen L., Gobba F. (2018). Solar radiation exposure and outdoor work: An underestimated occupational risk. Int. J. Environ. Res. Public Health.

[194] Roberts L., Jones G., Brooks R. (2018). Why Do You Ride?: A Characterization of Mountain Bikers, Their Engagement Methods, and Perceived Links to Mental Health and Well-Being. Front. Psychol..

[195] Rafie C., Ning Y., Wang A., Gao X., Houlihan R. (2018). Impact of physical activity and sleep quality on quality of life of rural residents with and without a history of cancer: Findings of the Day and night study. Cancer Manag. Res..

